# Rural-urban disparities in health outcomes, clinical care, health behaviors, and social determinants of health and an action-oriented, dynamic tool for visualizing them

**DOI:** 10.1371/journal.pgph.0002420

**Published:** 2023-10-03

**Authors:** William B. Weeks, Ji E. Chang, José A. Pagán, Jeffrey Lumpkin, Divya Michael, Santiago Salcido, Allen Kim, Peter Speyer, Ann Aerts, James N. Weinstein, Juan M. Lavista

**Affiliations:** 1 AI for Good Lab, Microsoft Corporation, Redmond, Washington, United States of America; 2 School of Global Public Health, New York University, New York, New York, United States of America; 3 Novartis Foundation, Basel, Switzerland; 4 Microsoft Research, Microsoft Corporation, Redmond, Washington, United States of America; 5 The Dartmouth Institute and Tuck School of Business, Dartmouth College, Hanover, New Hampshire, United States of America; 6 Kellogg School of Business, Northwestern University, Evanston, Illinois, United States of America; McGill University, CANADA

## Abstract

While rural-urban disparities in health and health outcomes have been demonstrated, because of their impact on (and intervenability to improve) health and health outcomes, we sought to examine cross-sectional and longitudinal inequities in health, clinical care, health behaviors, and social determinants of health (SDOH) between rural and non-rural counties in the pre-pandemic era (2015 to 2019), and to present a Health Equity Dashboard that can be used by policymakers and researchers to facilitate examining such disparities. Therefore, using data obtained from 2015–2022 County Health Rankings datasets, we used analysis of variance to examine differences in 33 county level attributes between rural and non-rural counties, calculated the change in values for each measure between 2015 and 2019, determined whether rural-urban disparities had widened, and used those data to create a Health Equity Dashboard that displays county-level individual measures or compilations of them. We followed STROBE guidelines in writing the manuscript. We found that rural counties overwhelmingly had worse measures of SDOH at the county level. With few exceptions, the measures we examined were getting worse between 2015 and 2019 in all counties, relatively more so in rural counties, resulting in the widening of rural-urban disparities in these measures. When rural-urban gaps narrowed, it tended to be in measures wherein rural counties were outperforming urban ones in the earlier period. In conclusion, our findings highlight the need for policymakers to prioritize rural settings for interventions designed to improve health outcomes, likely through improving health behaviors, clinical care, social and environmental factors, and physical environment attributes. Visualization tools can help guide policymakers and researchers with grounded information, communicate necessary data to engage relevant stakeholders, and track SDOH changes and health outcomes over time.

## Introduction

Despite overall improvements in mortality rates in the United States (US) between 2000 and 2019 (before the pandemic), disparities between rural and large metropolitan areas persist, and disparities in overall age-adjusted mortality rates tripled during that period [1]. The rural mortality penalty has increased, reducing lifespans in rural, as compared to urban, settings [2], with increasing rural-urban disparities in all-cause mortality having been shown among Medicare beneficiaries dually enrolled in Medicaid [3]. Residents of high-poverty rural counties face a particularly steep rural mortality penalty [4]. These rural-urban disparities in health outcomes span the age spectrum and disease states: along with differences in underlying health risks and behaviors, socioeconomic factors are associated with higher rates of the five leading causes of death [5], higher infant mortality rates [6], higher rates of cardiovascular disease mortality [7], and COVID-19-related deaths [8] in rural as compared to urban counties.

Socioeconomic variables have been shown to account for much of the mortality [9,10] and self-rated physical health status [11] differences between rural and urban populations. Possibly contributing to those disparities, the high relative use of preventable emergency department visits and hospitalization rates [12] and relatively low cancer screening rates [13] in rural settings suggests an unmet need for high-quality ambulatory care in rural areas. Nevertheless, among older Medicare beneficiaries, at the hospital referral region level primary care seems to be of similar quality and lower cost in rural as contrasted with urban settings after considering the role of local area deprivation [14]. This suggests that other factors, like social determinants of health (SDOH), may be contributing to these disparities more than clinical care quality.

SDOH are the non-medical factors that influence health outcomes: they encompass the conditions in which people are born, grow, work, live, and age as well as the wider set of forces and systems shaping the conditions of daily life [15]. SDOH can shape individuals’ health behaviors, which can then shape health outcomes [16]. The impact of SDOH as drivers of rural-urban (and racial) disparities across numerous health indicators in the US calls for a multi-sectoral approach to addressing SDOH in an effort to improve the health of the nation [17]. The distribution of economic prosperity among U.S. communities has undergone significant changes in recent decades, resulting in heightened inequality [18]. This has led to a growing interest in developing policies and resources that support both "places" and "people," particularly in underserved communities [19,20]. These policies recognize that socioeconomic conditions are significant determinants of health and that ameliorating SDOH disparities may improve health at the population level [21]. However, formulating an effective policy response requires identifying and targeting areas where interventions are most greatly needed, are achievable, and might have the largest and most sustained impact on health equity.

The aims of this study were to examine cross-sectional and longitudinal inequities in health, clinical care, health behaviors, and SDOH between rural and non-rural populations in 2015 and 2019 (the pre-pandemic era) and to develop and present a Health Equity Dashboard that can be used by policymakers and researchers to visualize and examine disparities across multiple SDOH domains and across time. While prior studies examined rural and urban health differences using data from County Health Rankings [22,23], to our knowledge, this is the first study to examine and visualize these differences across multiple years.

## Materials and methods

### Data

We sought to identify cross-sectional and longitudinal inequities in health, clinical care, health behaviors, and SDOH associated with rural status at the county level using data from County Health Rankings [24]. For 3,131 counties in the 50 US states and Washington, DC (wherein 325,711,203 people lived in 2019), we collected 33 county level attributes obtained from the 2015–2022 County Health Rankings across five health and SDOH domains: Health Outcomes, Clinical Care, Health Behaviors, Physical Environment, and Social and Economic Factors. We limited measures to those available for two time periods: approximately 2015 and approximately 2019.

**Table 1** provides the measure name, definition, orientation, periods of data collection, and year interval, across the five domains. **Table 2** shows the original sources from which County Health Rankings obtained these measures. We used the 2013 Urban-Rural Classification Scheme for Counties (based on the 2010 Census) [25] from the Centers for Disease Control and Prevention’s National Center for Health Statistics to classify counties as urban (codes 1 and 2), suburban (codes 3 and 4), or rural (codes 5 and 6).

**Table 1:** Measures collected, with domain, definition, orientation, periods obtained, and year interval between periods.

Domain	Measure name	Definition	Higher is…	First period	Second period	Year interval
**Health Outcomes**	Diabetes prevalence	Percentage of adults aged 20+ with diagnosed diabetes	Worse	2015	2019	4
	Fair or poor health	Age-adjusted percentage of adults in fair or poor health	Worse	2015	2019	4
	Frequent mental distress	Percentage of adults reporting 14+ days of poor mental health per month	Worse	2015	2019	4
	Frequent physical distress	Percentage of adults reporting 14+ days of poor physical health per month	Worse	2015	2019	4
	Life expectancy	Life expectancy at birth in years	Better	2015–17	2018–20	3
	Low birth weight	Percentage of live births that are <2500 grams	Worse	2010–16	2014–20	3
	Mentally unhealthy days	Age-adjusted average number of mentally unhealthy days in the last 30 days	Worse	2015	2019	4
	Physically unhealthy days	Age-adjusted average number of physically unhealthy days in the last 30 days	Worse	2015	2019	4
	Premature mortality	Age-adjusted number of deaths among residents under age 75 per 100,000	Worse	2015–17	2018–20	3
	Years potential life lost	Age-adjusted years of potential life lost before age 75 per 100,000 population	Worse	2015	2018–20	4
**Clinical Care**	Dental workforce	Ratio of population to dentists	Better	2015	2019	4
	Mammography screening rate	Percentage of female Medicare enrollees 65–74 that received annual mammogram screening	Better	2016	2019	3
	Mental health workforce	Ratio of population to mental health providers	Better	2015	2019	4
	PCP workforce	Ratio of population to primary care physicians	Better	2015	2019	4
	Preventable hospitalization rate	Preventable hospitalizations per 100,000 Medicare enrollees	Worse	2015	2019	4
	Uninsured	Percentage of population under age 65 that is uninsured	Worse	2015	2019	4
	Vaccinated	Percentage of fee-for-service Medicare enrollees that had an annual flu vaccine	Better	2016	2019	3
**Health Behaviors**	Chlamydia cases	Newly diagnosed chlamydia cases per 100,000 population (a measure of sexual risk)	Worse	2015	2019	4
	Excessive drinking	Percentage of adults reporting binge or heavy drinking	Worse	2015	2019	4
	Food index	Food environment index (0 to 10 point scale, 0 is worst)	Better	2015	2019	4
	Food insecurity	Percentage of population lacking adequate access to food	Worse	2015	2019	4
	Insufficient sleep	Percentage of adults reporting fewer than 7 hours of sleep on average	Worse	2015	2019	4
	Limited healthy food access	Percentage of population who are low-income and do not live close to a grocery store	Worse	2016	2018	2
	Obesity	Percentage of adults aged 20+ with a BMI≥30	Worse	2015	2019	4
	Physical inactivity	Percentage of adults aged 20+ reporting no leisure time physical activity	Worse	2015	2019	4
	Smokers	Percentage of adults who are current smokers	Worse	2015	2019	4
**Physical Environment**	Air quality	Average daily density of fine particulate matter in micrograms per cubic meter	Worse	2014	2018	4
	Severe housing problems	Percentage of households with at least 1 of 4 housing problems	Worse	2012–16	2014–18	5
**Social and Economic Factors**	Child food program participation	Percentage of children enrolled in public schools that are eligible for a free or reduced-price lunch	Worse	2012–16	2016–20	4
	Children in poverty	Percentage of population under age 18 living in poverty	Worse	2015	2019	4
	Deaths due to injury	Number of deaths due to injury per 100,000 population	Worse	2014–15	2018–19	4
	Income inequality	Ratio of household income at the 80th percentile to income at the 20th percentile	Worse	2015	2020	5
	Membership association rate	Number of membership associations per 10,000 population	Better	2011–15	2016–20	5

**Table 2:** Measures collected, with domain and original source of data that was compiled in County Health Reports.

Domain	Measure name	Original data source
**Health Outcomes**	Diabetes prevalence	United States Diabetes Surveillance System
	Fair or poor health	Behavioral Risk Factor Surveillance System
	Frequent mental distress	Behavioral Risk Factor Surveillance System
	Frequent physical distress	Behavioral Risk Factor Surveillance System
	Life expectancy	National Center for Health Statistics, Mortality Files
	Low birth weight	National Center for Health Statistics, Natality Files
	Mentally unhealthy days	Behavioral Risk Factor Surveillance System
	Physically unhealthy days	Behavioral Risk Factor Surveillance System
	Premature mortality	National Center for Health Statistics, Mortality Files
	Years potential life lost	National Center for Health Statistics, Mortality Files
**Clinical Care**	Dental workforce	Area Health Resource File
	Mammography screening rate	Mapping Medicare Disparities Tool
	Mental health workforce	Centers for Medicare and Medicaid Services, National Provider Identification
	PCP workforce	Area Health Resource File
	Preventable Hospitalization rate	Mapping Medicare Disparities Tool
	Uninsured	Small Area Health Insurance Estimates
	Vaccinated	Mapping Medicare Disparities Tool
**Health Behaviors**	Chlamydia cases	National center for HIV/AIDS, Viral Hepatitis, STD, and TB prevention
	Excessive drinking	Behavioral Risk Factor Surveillance System
	Food index	USDA Food Environment Atlas
	Food insecurity	Map the Meal Gap
	Insufficient sleep	Behavioral Risk Factor Surveillance System
	Limited healthy food access	USDA Food Environment Atlas
	Obesity	United States Diabetes Surveillance System
	Physical inactivity	United States Diabetes Surveillance System
	Smokers	Behavioral Risk Factor Surveillance System
**Physical Environment**	Air quality	Environmental Public Health Tracking Network
	Severe housing problems	Comprehensive Housing Affordability Strategy Data
**Social and Economic Factors**	Child food program participation	National Center for Education Statistics
	Children in poverty	Small Area Income and Poverty Estimates
	Deaths due to injury	National Center for Health Statistics, Mortality Files
	Income inequality	American Community Survey, 5-year estimates
	Membership association rate	County Business Patterns

### Analysis

Using Analysis of Variance (ANOVA), for both time periods we compared the health and SDOH measures across the rural-urban continuum (there were 436 urban counties, 729 suburban counties, and 1966 rural counties). Further, for each county, we calculated the change in values between 2015 and 2019. Finally, we calculated the ratio of values for rural to urban counties for the first and second data collection period (approximately 2015 and 2019) and provided an indication of whether the gap between the least and most prosperous counties was widening, narrowing, or staying the same. We used SPSS v 28 (released 2022, Armonk, NY: IBM Corporation) for all analyses.

We followed STROBE guidelines in writing the manuscript.

### Application and visualization

With the data we collected, we used Microsoft’s PowerBI platform to develop a Health Equity Dashboard that could be used by policymakers and researchers to examine disparities in single SDOH measures within domains, to develop their own indices of up to five measures across a single or multiple domains (calculated at the national or state level), and to examine the relationship between index values and county socio-demographic characteristics.

## Results

**Table 3** compares urban, suburban, and rural measures for 2019 data. For 25 of 33 measures, we found a statistically significant and progressive worsening of values when moving from urban to suburban to rural counties. For two measures (low birth weight and preventable hospitalization rate), rural values were worse than urban, but the pattern was not progressive (and not statistically significant in the case of low birth weight). For chlamydia cases (a behavioral risk factor that estimates the prevalence of unprotected sex), excessive drinking, air quality, severe housing problems, and membership association rates, values improved with increasing rurality. For insufficient sleep, rural values were better than urban values, but there was not a progressive (or a statistically significant) pattern.

**Table 3:** Results for the later data collection period (around 2019), across the rural-urban continuum.

Domain	Measure	Urban	Suburban	Rural
**Health Outcomes**	Diabetes prevalence	10.06	10.59	11.02
	Fair or poor health	18.12	19.91	21.43
	Frequent mental distress	14.59	15.78	16.34
	Frequent physical distress	12.15	13.42	14.20
	Life expectancy	78.32	77.22	76.46
	*Low birth weight#*	*8*.*04*	*8*.*27*	*8*.*23*
	Mentally unhealthy days	4.58	4.87	4.97
	Physically unhealthy days	3.92	4.26	4.46
	Premature mortality	351	391	429
	Years potential life lost	7512	8490	9223
**Clinical Care**	Dental workforce	58.40	54.55	41.74
	Mammography screening rate	42.90	44.15	41.00
	Mental health workforce	184	185	146
	PCP workforce	62.71	62.76	49.61
	Preventable hospitalization rate*	*40*.*55*	*39*.*10*	*40*.*91*
	Uninsured	9.88	11.06	12.71
	Vaccinated	49.09	47.52	39.87
**Health Behaviors**	**Chlamydia cases**	**450**	**473**	**388**
	Excessive drinking*	**19.43**	**19.17**	**18.95**
	Food index	8.25	7.59	7.23
	Food insecurity	10.50	12.64	13.83
	***Insufficient sleep#***	***36*.*90***	***36*.*98***	***36*.*75***
	Limited healthy food access	5.65	8.03	9.29
	Obesity	32.97	35.10	36.56
	Physical inactivity	27.27	29.15	31.49
	Smokers	17.55	19.52	21.29
**Physical Environment**	**Air quality**	**8.78**	**8.37**	**7.72**
	**Severe housing problems**	**14.72**	**13.99**	**12.81**
**Social and Economic Factors**	Child food program participation	44.81	52.34	57.11
	Children in poverty	13.28	17.39	20.35
	Deaths due to injury	79.78	85.29	97.36
	Income inequality	4.35	4.50	4.55
	**Membership association rate**	**8.96**	**10.45**	**12.43**

All ANOVA differences were statistically significant at p<0.001 except those marked with * which are p<0.05 and those marked # which are not statistically significant. Values in italics did not follow a progressive worsening of measure value with increasing rurality. Values in bold indicate measures where there was improvement in measure values with increasing rurality.

We conducted the same analysis using 2015 data (**Table 4).** Findings were similar: for 24 measures, there was a progressive and (save low birth weight) statistically significant worsening of values with increasing rurality; for four measures, rural values were worse than urban values, but there was no progressive pattern; for five measures, values improved with increasing rurality; and for chlamydia cases, rural values were better than urban ones, but there was no progressive pattern.

**Table 4:** Results for the earlier data collection period (around 2015), across the rural-urban continuum.

Domain	Measure	Urban	Suburban	Rural
**Health Outcomes**	Diabetes prevalence	10.78	11.37	11.92
	Fair or poor health	14.96	16.64	17.61
	Frequent mental distress	10.93	11.70	11.82
	Frequent physical distress	10.64	11.67	12.14
	Life expectancy	78.73	77.78	77.03
	Low birth weight#	7.97	8.14	8.14
	*Mentally unhealthy days*	*3*.*62*	*3*.*82*	*3*.*80*
	Physically unhealthy days	3.58	3.88	4.00
	Premature mortality	350	390	426
	Years potential life lost	6828	7721	8686
**Clinical Care**	Dental workforce	54.08	49.65	38.71
	*Mammography screening rate*	*40*.*63*	*42*.*11*	*39*.*04*
	Mental health workforce	138	138	112
	PCP workforce	62.97	61.90	50.49
	*Preventable hospitalization rate*	*52*.*50*	*52*.*30*	*64*.*43*
	Uninsured	10.06	11.12	12.79
	Vaccinated	45.82	44.75	37.72
**Health Behaviors**	***Chlamydia cases***	***384***	***404***	***342***
	**Excessive drinking**	**17.78**	**17.01**	**16.18**
	Food index	8.02	7.51	7.25
	Food insecurity	12.46	14.12	14.50
	**Insufficient sleep**	**34.19**	**33.56**	**32.60**
	Limited healthy food access	5.32	7.56	9.64
	Obesity	30.05	31.81	32.63
	Physical inactivity	23.31	24.76	26.59
	Smokers	16.15	17.59	18.38
**Physical Environment**	**Air quality**	**10.04**	**9.59**	**8.59**
	**Severe housing problems**	**15.27**	**14.50**	**13.28**
**Social and Economic Factors**	Child food program participation	45.38	52.36	56.40
	Children in poverty	17.32	22.07	25.01
	Deaths due to injury	67.30	73.60	89.33
	*Income inequality*	*4*.*40*	*4*.*56*	*4*.*53*
	**Membership association rate**	**9.69**	**11.49**	**15.59**

All ANOVA differences were statistically significant at p<0.001 except those marked # which are not statistically significant. Values in italics did not follow a progressive worsening of measure value with increasing rurality. Values in bold indicate measures where there was improvement in measure values with increasing rurality.

**Table 5** shows that diabetes prevalence, preventable hospitalization rate, and deaths due to injury all improved progressively with increasing rurality between the earlier and later period. For measures of years potential life lost, the PCP workforce, uninsurance, chlamydia cases, and children in poverty, rural values improved more than urban ones, though there was no progressive pattern. However, 22 measures worsened in a progressive fashion with increasing rurality and three measures worsened more in rural counties than in urban ones, but without a progressive pattern.

**Table 5:** Change in measure values between the earlier and later data collection period, across the rural-urban continuum.

Domain	Measure	Urban	Suburban	Rural
**Health Outcomes**	**Diabetes prevalence**	**-0.72**	**-0.78**	**-0.90**
	Fair or poor health	3.16	3.27	3.81
	Frequent mental distress	3.66	4.08	4.52
	Frequent physical distress	1.51	1.74	2.06
	Life expectancy	-0.41	-0.56	-0.58
	*Low birth weight*	*0*.*07*	*0*.*14*	*0*.*09*
	Mentally unhealthy days	0.97	1.05	1.16
	Physically unhealthy days	0.34	0.38	0.46
	Premature mortality	0.86	1.27	2.69
	***Years potential life lost***	***684***	***769***	***537***
**Clinical Care**	Dental workforce	4.32	4.90	3.03
	Mammography screening rate	2.27	2.04	1.96
	Mental health workforce	45.58	47.39	33.95
	*PCP workforce*	*-0*.*26*	*0*.*86*	*-0*.*88*
	**Preventable hospitalization rate**	**-11.95**	**-13.20**	**-23.52**
	***Uninsured***	***-0*.*18***	***-0*.*06***	***-0*.*08***
	Vaccinated	3.27	2.77	2.15
**Health Behaviors**	***Chlamydia cases***	***66*.*10***	***69*.*16***	***46*.*05***
	Excessive drinking	1.65	2.16	2.77
	Food index	0.22	0.07	-0.02
	Food insecurity	-1.96	-1.48	-0.67
	Insufficient sleep	2.71	3.42	4.14
	*Limited healthy food access*	*0*.*33*	*0*.*47*	*-0*.*35*
	Obesity	2.92	3.29	3.94
	Physical inactivity	3.96	4.39	4.90
	Smokers	1.40	1.93	2.91
**Physical Environment**	Air quality	-1.27	-1.23	-0.87
	Severe housing problems	-0.56	-0.50	-0.47
**Social and Economic Factors**	Child food program participation	-0.56	-0.03	0.71
	***Children in poverty***	***-4*.*04***	***-4*.*68***	***-4*.*67***
	**Deaths due to injury**	**12.48**	**11.69**	**8.03**
	*Income inequality*	*-0*.*05*	*-0*.*07*	*0*.*01*
	Membership association rate	-0.73	-1.04	-3.15

Values in italics did not follow a progressive worsening of measure value when moving to increasing rurality. Values in bold indicate measures where there was improvement in measure values with increasing rurality.

**Table 6** shows the ratio of rural to urban values in the earlier and later periods. During that time, the rural-urban gap widened for 15 measures, narrowed for 12 measures, and did not change for 6 measures. Gaps tended to widen in the health outcomes and health behaviors domains and tended to narrow in the clinical care domain.

**Table 6:** Ratios of values in the rural to urban counties in 2015 and 2019 and an indication of whether the rural urban-gap narrowed, widened, or did not change.

Domain	Measure	Higher is..	Ratio of rural to urban values		Between 2015 and 2019, the rural-urban gap…
			2015	2019	
**Health Outcomes**	Diabetes prevalence	Worse	1.11	1.10	Narrowed
	Fair or poor health	Worse	1.18	1.18	Did not change
	Frequent mental distress	Worse	1.08	1.12	Widened
	Frequent physical distress	Worse	1.14	1.17	Widened
	Life expectancy	Better	0.98	0.98	Did not change
	Low birth weight	Worse	1.02	1.02	Did not change
	Mentally unhealthy days	Worse	1.05	1.08	Widened
	Physically unhealthy days	Worse	1.12	1.14	Widened
	Premature mortality	Worse	1.22	1.22	Did not change
	Years potential life lost	Worse	1.27	1.23	Narrowed
**Clinical Care**	Dental workforce	Worse	0.72	0.71	Narrowed
	Mammography screening rate	Better	0.96	0.96	Did not change
	Mental health workforce	Worse	0.81	0.79	Narrowed
	PCP workforce	Worse	0.80	0.79	Narrowed
	Preventable hospitalization rate	Worse	1.23	1.01	Narrowed
	Uninsured	Worse	1.27	1.29	Widened
	Vaccinated	Better	0.82	0.81	Narrowed
**Health Behaviors**	Chlamydia cases	Worse	0.89	0.86	Narrowed
	Excessive drinking	Worse	0.91	0.98	Widened
	Food index	Better	0.90	0.88	Narrowed
	Food insecurity	Worse	1.16	1.32	Widened
	Insufficient sleep	Worse	0.95	1.00	Widened
	Limited healthy food access	Worse	1.81	1.64	Narrowed
	Obesity	Worse	1.09	1.11	Widened
	Physical inactivity	Worse	1.14	1.15	Widened
	Smokers	Worse	1.14	1.21	Widened
**Physical Environment**	Air quality	Worse	0.86	0.88	Widened
	Severe housing problems	Worse	0.87	0.87	Did not change
**Social and Economic Factors**	Child food program participation	Worse	1.24	1.27	Widened
	Children in poverty	Worse	1.44	1.53	Widened
	Deaths due to injury	Worse	1.33	1.22	Narrowed
	Income inequality	Worse	1.03	1.05	Widened
	Membership association rate	Better	1.61	1.39	Narrowed

**Fig 1:**
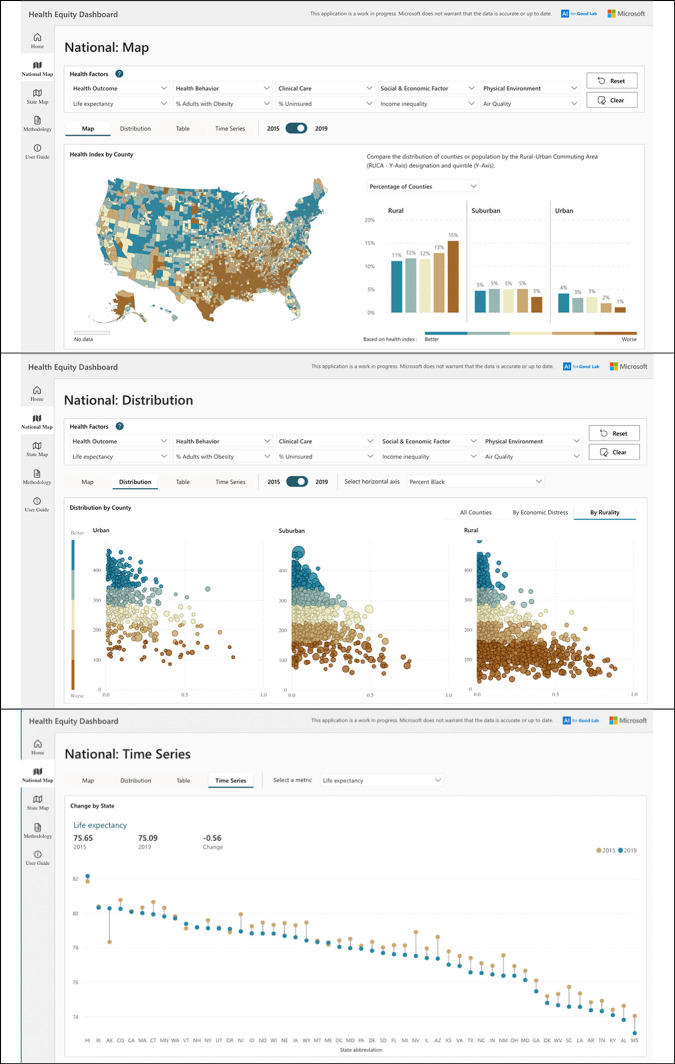
A national map view of the Health Equity Dashboard, showing: the 2019 distribution in quintiles of a national index formed from five measures (life expectancy, percentage of the adult population that is obese, percentage of the population that is uninsured, income inequality, and air quality) and the percentage of rural, suburban, and urban counties with index values in the best to worst quintiles (top); the county-level distribution of that index across urban, suburban, and rural county designations (middle); and a comparison of the 2015 and 2019 values of one measure (percentage of the population in fair or poor health) at the state level (bottom). The Health Equity Dashboard tool is publicly available at: aka.ms/healthequity. The base layers for the maps are Shapefiles from the US Census TIGER file repository.

**Fig 1** shows an example of how nationally oriented policymakers and researchers might use the Health Equity Dashboard (publicly available at aka.ms/healthequity). The user could create a map of nationally calculated index values (in this example, composed of five equally weighted, 2019 measures (life expectancy, percentage of adults with obesity, uninsurance rate, income inequality, and air quality)) at the county level (**Fig 1, top**), explore the distribution of index values (in quintiles) across the rural-urban continuum (**Fig 1, middle**), and examine a measure’s mean value at the state level, over time (**Fig 1, bottom**). Such users would discover that Los Alamos County (rural) in New Mexico had the best index score in the nation and Brooks County, Texas (also rural) had the worst index score. They would discover a worsening of index scores with increasing rurality and in counties with higher proportions of Blacks. In the time series comparison, they would find that Mississippi had the highest percentage of adults in fair or poor health (26.66%) while Connecticut had the lowest percentage (13.98%); further, they would discover that between 2015–2019, the percentage of adults in fair or poor health increased in every state, the most in Florida (from 16.44% to 22.82%) and the least in Massachusetts (from 13.26% to 14.59%).

**Fig 2:**
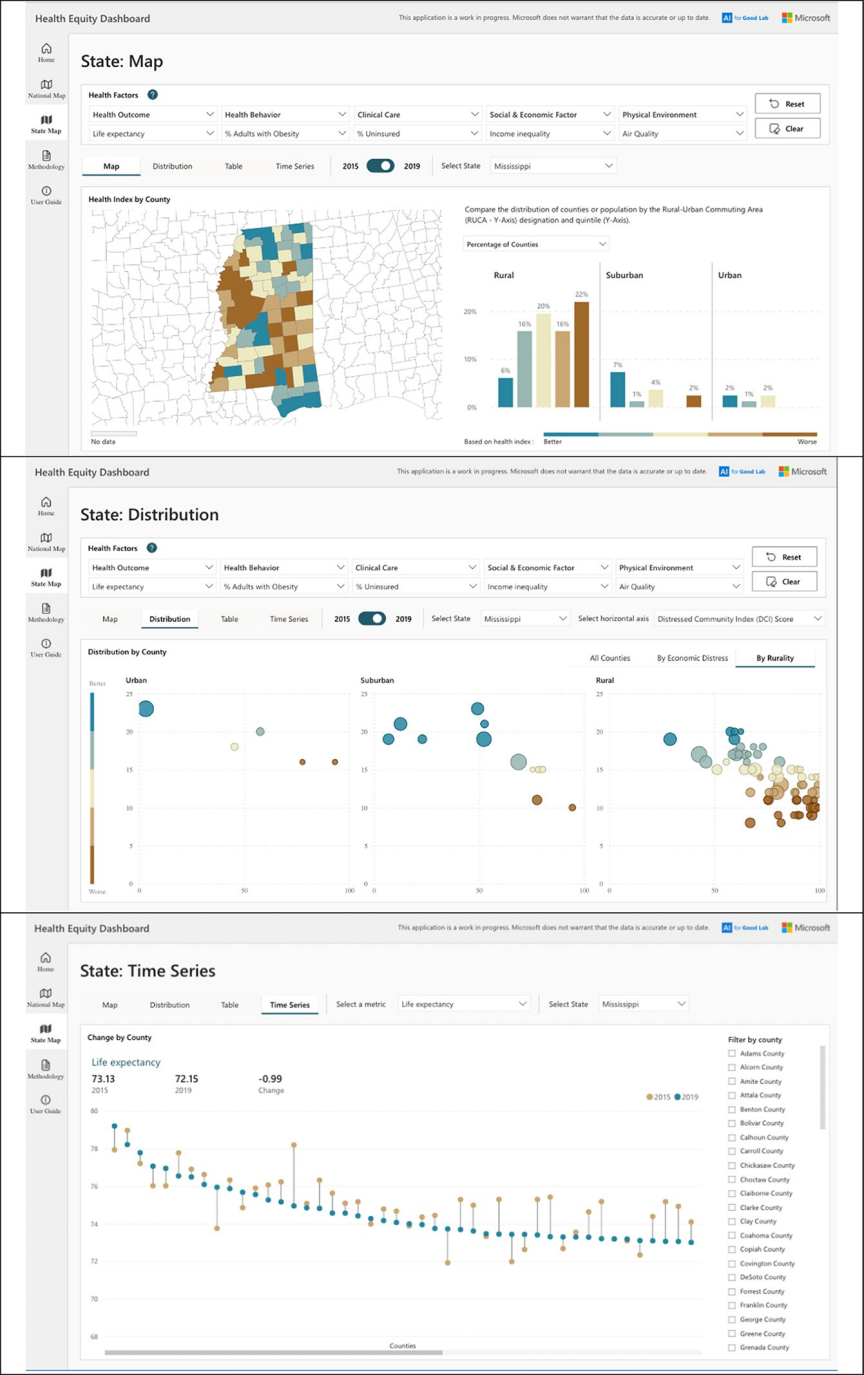
A state-level view of the Health Equity Dashboard, showing, for Mississippi: the 2019 distribution in quintiles of a Mississippi-specific generated index formed from five measures (life expectancy, percentage of the adult population that is obese, percentage of the population that is uninsured, income inequality, and air quality) and the percentage of rural, suburban, and urban counties with index values in the best to worst quintiles (top); the county-level distribution of that index across urban, suburban, and rural county designations (middle); and a comparison of the 2015 and 2019 values of one measure (life expectancy at birth) at the state level (bottom). The Health Equity Dashboard tool is publicly available at: aka.ms/healthequity. The base layers for the maps are Shapefiles from the US Census TIGER file repository.

**Fig 2** shows an example of how the dashboard might be used to differentiate counties with greater relative need, within a state. For instance, when recalculating the previously-defined index at a state level, policymakers or researchers interested in Mississippi–a state in which virtually every county was in the worst health index quintile from a national perspective–could examine relative differences in index values within their state (**Fig 2, top**), while still noting the rural-urban disparities (**Fig 2, middle**), and appreciating county-level changes in chosen metrics (in this case, life expectancy) over time (**Fig 2, bottom**). A policymaker or researcher interested in Mississippi would discover that their 2019 measure index values indicated that DeSoto County (a prosperous, urban county with a population of 182,256) had the best index score in Mississippi (despite being in the middle quintile for the nation) while Covington County (an economically distressed rural county with a population of 18,810) had the worst index score in Mississippi. Further, they would discover that the prevalence of worst quintile index scores was highest in rural counties which also were much more likely to be experiencing economic distress. Finally, they could see that, between 2015 and 2019, life expectancy at birth decreased from 73.13 to 72.15 years across counties, increased for 15 Mississippi counties, ranged from 73.00 in Prentiss County to 79.21 in Lamar County in 2019, and ranged from 71.92 in Attala County to 78.97 in Rankin County in 2015.

**Fig 3:**
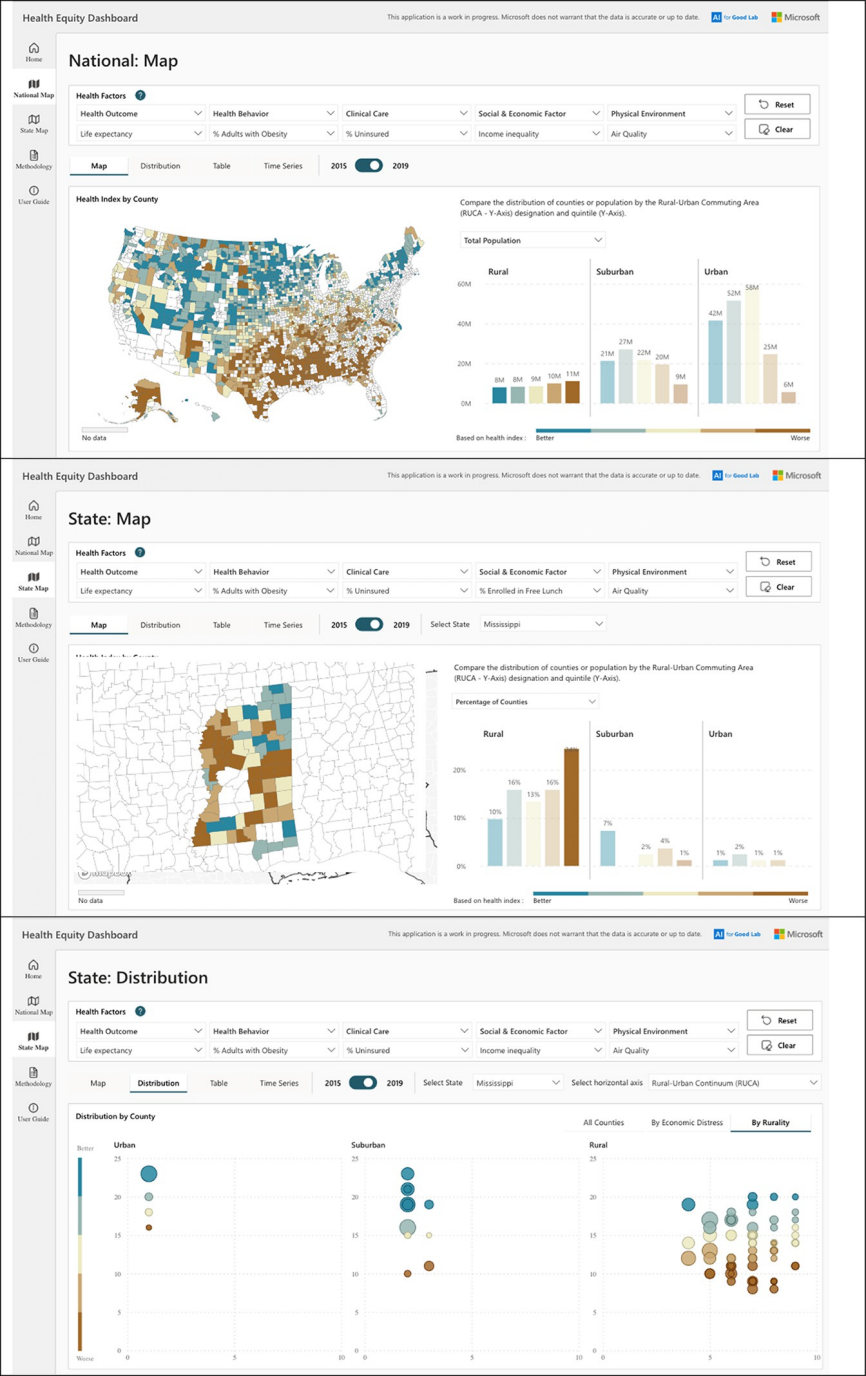
Examples of how policymakers or researchers might use the Health Equity Dashboard, by showing only rural counties and the distribution of nationally-defined index quintiles within rural counties at the national level (top), by showing only rural counties and the distribution of state-defined index quintiles within rural counties at the state level (middle), or by comparing nationally-defined index values for counties across a measure of local economic prosperity and 2013 rural-urban continuum code assignments. The Health Equity Dashboard tool is publicly available at: aka.ms/healthequity. The base layers for the maps are Shapefiles from the US Census TIGER file repository.

Finally, should a policymaker or researcher want to examine and compare index or measure values only for rural counties, they could select ‘rural’ on national (**Fig 3, top)** or state (**Fig 3, middle**) maps, to highlight only rural counties. There, from a national perspective, they might find a high proportion of rural counties in the Midwest, worse index scores within rural counties across the southeastern United States, and that about 11 million people lived in rural counties in the worst index quintile (compared to about 9 million in suburban counties and about 6 million in urban counties). Further, nationally or within a state, they might examine relationships between index scores and, 2013 Rural-Urban Continuum Codes [26], finding, in Mississippi, worse overall scores–but a broader range of scores–in counties coded with Rural-Urban Continuum Codes of six, seven, or eight (**Fig 3, bottom**), with Neshoba County having the worst index score among counties coded seven and Itawamba County having the best.

## Discussion

We found that rural counties overwhelmingly had worse measures of health and SDOH at the county level. With few exceptions, many of the measures we examined were getting worse between 2015 and 2019 in all counties; in addition, measures generally got relatively worse in rural counties, resulting in the widening of rural-urban disparities in these measures during this period. In the health behaviors domain, while chlamydia cases were lower and increasing at a slower rate in rural settings, the rural advantage in excessive drinking is diminishing. The good news for rural dwellers is in the physical environment realm, where air quality is better and housing problems are fewer. While the membership association rate is higher in rural settings, that advantage is diminishing as well.

While our findings may not be unexpected, the opportunity to examine numerous SDOH measures together, across time, and through index development may offer policymakers and researchers an opportunity to consider where best to focus efforts and which factors to focus on, across the country or within a state. Further, the ability to consider the potential market–as represented by population distributions and numbers of counties–might inform policymakers or researchers interested in health equity on the overall impact proposed programs might have.

Our study has several limitations. First, we used one coding system to categorize counties into rural, suburban, or urban.

There are multiple systems to designate counties and places within a rural-urban continuum [27], and different ways to interpret what “rural health” means [28]. Findings may be different when using different rural-urban continuum classification systems. Second, our results are derived from data in two relatively close time periods; studies of different time periods may have different results. Importantly, we evaluated periods before the COVID-19 pandemic; reports suggest that economic and health inequities have increased since COVID-19 began [29]. Therefore, our results might underestimate current inequities. Third, measures are not adjusted for local demographic factors that may impact measure values. For example, Blacks are more likely than Whites to have diabetes [30], lower life expectancy [31], and low birth weight babies [32]. To the extent that racial disparities are conflated with the rural-urban disparities we found, our analysis is limited. However, while demographic factors may be partially explanatory [33], they offer policymakers no pragmatic solutions: changing the demographic makeup of a county cannot be a reasonable policy platform. Finally, our findings are associative and not causative.

Despite these limitations, our findings highlight the need for policymakers to prioritize rural settings for interventions designed to improve health outcomes, likely through improving health behaviors, clinical care, social and environmental factors, and physical environment attributes. Timely, accurate, and high-quality data are a critical component of public health decision making [34]. Data visualization tools can help the effective delivery and translation of data, thereby engaging key stakeholders and prompting action [35,36]. By leveraging these tools, policymakers can make more informed decisions that are grounded in objective evidence, ultimately leading to better outcomes for all stakeholders. As all policy decisions have population health implications [37], interventions should be evaluated for return on investment to population health and reduction of rural-urban disparities, as well as any other policy goals. Tools like the Health Equity Dashboard (publicly available at aka.ms/healthequity) can facilitate those evaluations. Hopefully, by guiding policymakers with grounded information in a way that can be personalized to a community’s interest and consumed, shared, and tracked visually, over time, policies can be developed and focused to measurably improve population health in areas where the greatest health inequities exist and those with the greatest unmet social needs reside.
